# Non-Cohesive Gold Fillings

**Published:** 1903-05-15

**Authors:** G. W. Haskins


### NON-COHESIVE GOLD FILLINGS.

Proximal cavities in bicuspids and molars are prepared with a step in the occlusal surface, with broad base at the gingival portion and extending lingually and buccally for the prevention of future decay. The lingual and buccal walls of the cavity need not be at right angles to the axial wall, but the gingival wall should be, and should have a decided retention groove cut in it, which grooving should extend up the lingual and buccal walls, but should be of slight depth and cease entirely before passing the occlusal third of the cavity wall. The step in the occlusal portion of the tooth should have a very positive retentive shape and of such depth that the dislodging strain of mastication will come upon the dentine and not upon the enamel. The axial wall should have no irregularities upon its surface, but be made flat with a wide chisel so the cylinder of gold will have nothing to interfere with its passing readily to place. The enamel margins should be beveled.

In occlusal cavities the walls should be parallel with the long axis of the tooth, with slight retention grooves and the enamel margins beveled. The inverted cone bur is a useful instrument for such cavities and gives all the retentive shape needed, in addition leaving the cavity with a flat pulpal wall at the bottom.

The gold should be a positively non-cohesive foil; otherwise, all values to be gained by its use are lost. I find use for but two forms of gold foil—absolutely non-cohesive and the most cohesive I can find. For many years I used Abbey's No. 4, and have since used Williams' No. 3 non-cohesive foil.

The amount of gold in each sheet of the latter seems more frequently to be just the quantity needed than is the case with the No. 4. Never use less than half a sheet at a time, and from that up to from two to three sheets, according to the size of the cavity; the gold is first folded carefully into a ribbon, being sure to bring the folded edges evenly together. In proximal cavities the width should be as near the mesio-distal depth of the cavity as can be, having it a trifle wider rather than the least bit too narrow. For occlusal cavities the ribbon should be one-half wider than the depth of the cavity, to allow for surface condensation. The ribbon can then be rolled upon a broach in to a cylinder, or with the pliers folded into an ellipse or cushion whose major axis is in length twice the width of the cavity. The ellipse is better than the cylinder in some cases, for the reason that it has not the dense center of the latter.—G. W. Haskins, in *Review*.

